# Symptomatic Correlates of Vitamin D Deficiency in First-Episode Psychosis

**DOI:** 10.1155/2019/7839287

**Published:** 2019-05-02

**Authors:** Ricardo Coentre, Inês Canelas da Silva

**Affiliations:** ^1^Department of Psychiatry, Hospital de Santa Maria, Centro Hospitalar Universitário Lisboa Norte EPE/Faculdade de Medicina, Universidade de Lisboa, Lisboa, Portugal; ^2^Department of Psychiatry, Hospital Vila Franca de Xira, Vila Franca de Xira, Portugal

## Abstract

Previous studies indicate that low levels of vitamin D are associated with increased severity of psychiatric symptoms in chronic multiepisode psychosis (MEP). We aimed to compare vitamin D levels between first-episode psychosis (FEP) and MEP and to investigate the correlations between vitamin D levels and symptoms in FEP patients. The participants were adults aged 18-45 years who presented with affective and non-affective FEP to an early intervention team in Portugal. Depression was assessed using the Beck Depression Inventory, and positive and negative symptoms and general psychopathology were measured with the Positive and Negative Syndrome Scale. Blood samples were analyzed for 25-hydroxyvitamin D (25OHD). Thirty-three patients completed the study in the FEP group and 33 in the MEP group. FEP patients had low levels of 25OHD (18.16 ± 7.48 ng/mL), with no significant differences from MEP patients. Low 25OHD was significantly correlated with high severities of depressive (r=-0.484, p=0.004) and negative (r=-0.480, p=0.005) symptoms as well as general psychopathology (r=-0.569, p=0.001) in FEP patients. Multiple regression revealed an inverse association between general psychopathology and vitamin D level (p=0.027). More investigation of the association of vitamin D and schizophrenia is needed, namely, using a nonpatient control group and trying to clarify possible causality between vitamin D and psychiatric symptoms.

## 1. Introduction

Vitamin D deficiency is traditionally associated with musculoskeletal consequences [1]. In recent years vitamin D has been associated with psychiatric disorders [2].

Vitamin D is primarily produced by skin exposure to ultraviolet B sunlight with diet contributing with a small percentage (a maximum of 20%) [3]. Vitamin D levels vary seasonally with lower levels occurring during the late winter and early spring that reflect the levels of ambient sunlight [4]. People with pigmented skin need more sunlight to produce vitamin D, and lower levels of vitamin D are observed in Black and Asian populations [5]. Serum 25-hydroxyvitamin D (25OHD) is considered the optimal vitamin D status indicator [6], and its hydroxylation in the kidneys produces 1,25-dihydroxyvitamin D (1,25(OH)2D), which is the active metabolite. Approximately 85-90% of 25OHD and 1,25(OH)2D are transported in the circulation by vitamin D-binding protein, which is a water-soluble carrier protein. Only 1% of the metabolites bind weakly to albumin to freely circulate in the bloodstream, and it is the free fraction of the hormone that is the biologically active component [7].

Vitamin D levels in the general population are higher in men than in women, they decrease with increasing age [8], and because vitamin D is fat soluble and stored in adipose tissue, those with obesity have lower circulating levels [9]. There is also a relationship between low levels of vitamin D and tobacco [10], because tobacco increases hepatic cytochrome enzyme activity and consequently alters vitamin D metabolism [11].

Vitamin D is involved in several brain processes, including neurodevelopment, neurotrophic activities, and growth factor regulation, and vitamin D also has a neuroprotective role [12]. Several studies have suggested that vitamin D levels are related to the development of psychosis [2]; namely, a correlation between low early life vitamin D levels and schizophrenia has been suggested [13, 14]. There is also evidence of increased prevalence of schizophrenia in urban environments, among dark-skinned migrants, among those with winter births and those at higher latitudes [15, 16]. It has been hypothesized that vitamin D could be responsible for these results [15]. Vitamin D deficiency could be a modifiable risk factor for psychotic disorders. Published studies have mainly included patients with established and chronic psychotic disorders and populations living at high latitudes with significant seasonal periods [2]. Few studies exist regarding the early phases of psychosis and involving those living in low/medium latitude countries. Indeed, the published research does not clarify the main question: is vitamin D deficiency the cause and/or consequence of psychosis [17]?

Several studies have demonstrated an inverse correlation between vitamin D levels and positive, negative and depressive symptoms in chronic psychosis [17, 18]. Few studies have researched vitamin D in first-episode psychosis (FEP) patients [19–23]. Only two studies have evaluated the association between vitamin D and symptoms in FEP. In Singapore, Yee et al. (2016) found an association between low levels of bioavailable vitamin D and negative symptoms in FEP patients [23]. Additionally, in North Carolina, USA, Graham et al. (2015) found that a greater severity of negative symptoms was correlated with lower vitamin D status in first-episode schizophrenia patients [20]. Also, some published studies indicate that anticonvulsant medication could have a negative effect on vitamin D levels [24].

The aims of the present study were as follows: (1) to investigate vitamin D levels in affective and non-affective FEP in a medium latitude country (40° N) that provides significant sunlight exposure during the entire year, (2) to compare vitamin D levels in FEP subjects with those in chronic multiepisode psychosis (MEP) subjects, and (3) to examine the relationship between vitamin D levels and symptom severity (positive, negative, and depressive symptoms and general psychopathology) in FEP. We hypothesized that FEP patients would have low levels of vitamin D that do not differ from those in MEP patients, which would reflect the early presence of vitamin D deficiency in psychotic disorders. We also hypothesized that FEP patients would exhibit negative correlations of vitamin D with general, negative, and depressive symptoms similar to the results that have been found in previous studies of MEP.

## 2. Material and Methods

### 2.1. Subjects

All participants were part of a larger prospective study of FEP at the First-Episode Psychosis Program (PPEP) in Vila Franca de Xira, Portugal. The PPEP is a specialized early intervention program that provides medical and psychosocial treatment and follow-up for affective and non-affective FEP patients to a catchment area with a population of 245,000. Patients aged 18 to 45 from the local catchment area who were consecutively admitted between January 2016 and March 2017 were eligible for participation in the study. The exclusion criteria were the following: a primary diagnosis of substance abuse or dependence, organic psychosis, presence of major medical/neurological disease, and an inability to understand and complete the assessments.

The subjects were provided full information about the nature and purpose of the study and were informed of the possibility of terminating their participation at any time. This study was conducted in accordance with the ethical principles contained in the Declaration of Helsinki. The research protocol was approved by Hospital Vila Franca de Xira Ethics Committee, and written informed consent was obtained from all participants.

### 2.2. Methods

At program entry, a trained consultant psychiatrist collected detailed sociodemographic and clinical data as part of the initial routine clinical care of FEP patients. The clinical data included the duration of untreated psychosis (DUP), depressive symptoms, positive symptoms, negative symptoms, general psychopathology, and adherence to medication.

The duration of untreated psychosis was determined using the Nottingham Onset Schedule (NOS) [25] and was defined as the period of time from the onset of psychotic symptoms to treatment with antipsychotic medication. The diagnoses were established using the Operational Criteria Checklist for Psychotic Illness and Affective Illness (OPCRIT+) [25, 26]. The OPCRIT+ is a checklist that includes items related to psychiatric history and psychopathology. The checklist ratings are entered into the OPCRIT+ software, which generates a diagnosis for the main categories of affective and psychotic disorders as defined according to major classification systems including the DSM-IV. The OPCRIT+ has been demonstrated to have good reliability when used by different raters. The rater was an experienced consultant psychiatrist who was trained in the use of the OPCRIT+.

Positive and negative symptoms and general psychopathology were assessed with the Positive and Negative Syndrome Scale (PANSS) [27]. This scale is composed of three subscales that evaluate positive symptoms, negative symptoms, and general psychopathology in schizophrenia. Each item is scored from 1 (absent) to 7 (extreme). The positive subscale includes 7 items about positive symptoms (e.g., delusions, hallucinatory behavior, and hostility), and the total score varies between 7 and 49. The negative subscale includes items related to negative symptoms (e.g., blunted affect, poor rapport, or difficulty in abstract thinking), and the total score varies between 7 and 49. The general psychopathology subscale includes a variety of items related to psychopathology other than positive and negative symptoms, including anxiety, depression, somatic concern, lack of judgment and insight, and poor impulse control. The total score for the general psychopathology subscale varies between 16 and 112.

Depressive symptoms were evaluated with the Beck Depression Inventory (BDI) [28]. This instrument is a 21-question, multiple-choice, and self-report inventory that is used to evaluate the incidence and severity of depression symptoms with a total score between 0 and 63. The version of the BDI scale that had been translated into the Portuguese language and validated for the Portuguese population was used [28, 29]. To examine attitude and adherence behavior towards antipsychotic treatment, the Medication Adherence Rating Scale (MARS) was applied [30]. This scale has 10 yes or no questions about medication and behavior toward medication and results in a total score that ranges from 0 to 10; higher scores indicate better adherence. The Global Assessment of Functioning Scale (GAF) was used to evaluate general functioning [31]. The GAF subjectively evaluates social, occupational, and psychological functioning, and the final score ranges from 1 (severely impaired) to 100 (extremely high functioning).

Blood collection was performed between 8.00 and 9.00 am upon entry to the study. Blood was withdrawn by venipuncture into tubes containing SST for the collection of the serum. The optimal vitamin D status indicator, i.e., 25-hydroxyvitamin D (25OHD), was obtained by chemiluminescent immunoassay. The 25OHD levels were classified as sufficient if the value was ≥30 ng/mL and insufficient if the value was <30 ng/mL.

### 2.3. Statistical Analysis

The data were analyzed using IBM SPSS Statistics version 24. The descriptive statistics are reported as the means and standard deviations for continuous measurements and as the frequencies and proportions for categorical measurements. Demographic and clinical variables were compared between groups with independent Student's t-tests or chi-squared tests (or Fisher's exact tests) for continuous and categorical variables, respectively. Correlation analyses were performed with Pearson correlation coefficients. The Kolmogorov-Smirnov test was used to confirm the normal distributions of the variables. Multiple linear regression was used to assess the relationships between the independent variables (the depressive, positive, and negative symptoms and general psychopathology) and the dependent variable (25-hydroxyvitamin D). Clinical assessments were performed on the same day as blood sampling. All evaluations were performed when the clinical picture of FEP had sufficient stability to warrant collaboration by the patient. A p value <0.05 was considered to be statistically significant.

## 3. Results

### 3.1. Comparison of the Demographics and Vitamin D Levels between the Samples

Overall, 33 patients with FEP and 33 patients with MEP were enrolled. Only 1 patient in FEP group was excluded because the diagnosis of organic psychosis was made. Descriptions of the participants and comparisons between the two groups are summarized in Table 1. There were no significant differences in gender, ethnicity, tobacco use, diagnoses, body mass index (BMI), or blood collection season between the two groups.

As expected, the FEP sample was significantly younger than the MEP sample (mean 31.21 vs. 41.15 years; p<0.001). The mean vitamin D levels were similar between the two groups (mean 18.16 ± 7.48 ng/mL for the FEP group and 16.20 ± 11.29 ng/mL for the MEP group; p=0.409). On the FEP group, 25 (75.75%) patients were medicated with antipsychotic alone, 6 (18.18%) with antipsychotic plus antidepressant, and 2 (6.06%) with mood stabilizer/anticonvulsivant plus antipsychotic. There was no evidence of the effect of medication on vitamin D levels, including anticonvulsivant medication. Only two patients on FEP group (mean vitamin D level 14.45 ng/mL) and four on MEP group (mean vitamin D level: 20.95 ng/mL) were prescribed anticonvulsants, all with valproic acid.

### 3.2. Correlation Analysis

The 25OHD level was inversely correlated with negative symptom (r=-0.480, p=0.005) and general psychopathology (r=-0.569, p=0.001) scores on the PANSS and with the depressive symptom (r=-0.484, p=0.004) scores as evaluated with the BDI in the FEP group. No correlation was found between 25OHD level and positive symptoms (Table 2) in the FEP sample. Also, no significant correlations were found between vitamin D levels and DUP, MARS, and GAF scores. Multiple linear regression analysis examining the associations of positive, negative, general psychopathology, and depressive symptoms (independent variables) with 25-hydroxyvitamin D (dependent variable) revealed an inverse correlation with general psychopathology (p=0.027).

## 4. Discussion

This study examined vitamin D levels in minimally treated FEP patients in comparison with MEP in a medium latitude country. We also assessed the clinical symptoms in FEP and the correlations of vitamin D concentrations with positive, negative, and depressive symptoms and general psychopathology. Our main findings were the following: (1) we found no significant difference in vitamin D levels between the FEP and MEP samples; (2) even in a country with a significant exposure to sunlight, both the FEP and MEP groups exhibited low levels of vitamin D; and (3) vitamin D levels were inversely correlated with general psychopathology in FEP.

To our knowledge, only two other studies have examined the correlation between vitamin D and psychotic and/or affective symptoms in FEP. Graham et al. (2015) found that greater severities of negative symptoms are correlated with lower vitamin D levels but not with depressive or overall symptoms in first-episode schizophrenia patients in North Carolina, USA [20]. There was no significant difference in the mean vitamin D level between patients with schizophrenia and healthy controls [20]. More recently, Yee et al. (2016) found an association between low bioavailable vitamin D and negative symptoms in FEP in Singapore [23]. We did not find correlations in the multiple linear regression between vitamin D and negative symptoms as reported in these two previously mentioned studies. It is possible that our results were limited by the lower levels of vitamin D compared with those observed in the study published by Graham and colleagues [20].

Moreover, studies of chronic schizophrenia patients revealed associations of vitamin D with depressive and negative symptoms. Positive associations of low levels of vitamin D with negative [17, 18, 31, 32] and depressive symptoms [18] have been found in chronic psychotic patients, particularly those with schizophrenia.

The results of our research demonstrate that vitamin D deficiency is present in psychotic disorders even in the early phases of the disorder and in countries with regular sunlight exposure throughout the year. The main question persists: is vitamin D deficiency the cause and/or consequence of the psychotic disorder? Our results indicate that vitamin D could have an etiological role in some psychopathological symptoms of psychosis, and thus its deficiency is present in the early stages of the disorder. For example, due to the neuroprotective function of vitamin D that prevents oxidative stress in the central nervous system, there is a hypothesis that suggests that oxidative stress resulting from vitamin D deficiency causes negative symptoms due to an imbalance in glutamate-GABA responses [33, 34]. Vitamin D is also associated with depression because it is a regulator of serotonin synthesis [35]. In the current research, we only found a negative correlation between vitamin D status and the general psychopathology subscale of the PANSS. This subscale includes a variety of psychiatric symptoms, such as depressive, anxious, and physical symptoms. Contrary to the previously mentioned studies, we did not find any significant correlation between vitamin D and negative symptoms in FEP. Independently of the type of symptoms found to be associated, vitamin D deficiency has been found to be associated with severe psychiatric symptoms in all studies, including ours. Some authors also speculate that patient behaviors that result from negative symptoms and are present in all phases of the disorder, including the prodrome, originate from long periods of time spent indoors without sunlight exposure that result in low levels of vitamin D [36].

Recently a published article from Eyles and colleagues studied the association between neonatal vitamin D status and risk of schizophrenia in a large Danish case-control study, including 2602 neonates [37]. Results showed that those in the lowest quintile (<20.4noml/L) had an increased risk of schizophrenia. This is an argument in line with the neurodevelopment hypothesis of schizophrenia, where vitamin D deficiency in early phases of life could also represent a risk factor for schizophrenia later.

Research indicates the existence of a preliminary evidence that certain vitamin and mineral supplements may reduce psychiatry symptoms in some people with schizophrenia [38]. This includes vitamin B supplementation, but not vitamin D yet. Based in our results, vitamin D deficiency could represent a modifiable factor of psychopathology in psychosis and could condition the clinical picture. Vitamin D as an add-on treatment could be recommended for patients with low levels. Evidence in favor of this treatment might soon be provided by the final results of the two trials (ClinicalTrials.gov Identifiers: NTC01759485 and NCT01169142). One is a randomized, double blind placebo-controlled trial of which the main objective is to evaluate the effect of vitamin D supplementation on the mental states of clozapine-treated patients with chronic schizophrenia and vitamin D deficiency (20-30 ng/mL) and the relation of disease severity with serum vitamin D level. The second study is a randomized open-label trial that includes patients with schizophrenia and schizo-affective disorder and low levels of vitamin D (<30 ng/mL) who will be treated with vitamin D supplementation. Unfortunately, there is no ongoing trial that includes first-episode psychosis patients. Currently, there is not sufficient evidence to support vitamin D screening and supplementation for psychotic patients.

Several limitations of our study exist. First, the relatively small sample size could limit the generalization of the data, even though our sample is larger than those of previously published papers in this field of knowledge. Second, the cross-sectional design precludes the ability to provide evidence of a causal relationship between low levels of vitamin D and symptoms in FEP. Third, we did not control for confounding factors (e.g., daily calcium intake or sun exposure), which might have influenced our findings. Fourth, the self-report nature of some of the scales and data, namely, the socially undesirable behaviors, such as cannabis use or tobacco smoking, may be subject to reporting bias. Moreover, the information regarding the self-reported psychiatric symptoms (e.g., depressive symptoms) may not have been accurate. Fifth, we did not include a healthy control group, which would have helped to differentiate whether low levels of vitamin D are mainly found in patients with psychiatric disorders or if they are also found in the general population. Sixth, the generalization of our findings to countries of different latitudes and consequently different levels of sun exposure and different vitamin D levels must be performed cautiously.

## 5. Conclusion

In conclusion, there is evidence demonstrating that there are low levels of vitamin D in psychotic disorders beginning in the early stages and that vitamin D could have a pathophysiological role in psychosis. The results of research, including ours, demonstrate the correlation between low levels of vitamin D and high severity of psychiatric symptoms in all stages of psychotic disorders. There are ongoing trials that are evaluating vitamin D as a possible adjuvant treatment in chronic multiepisode psychotic patients with low levels of vitamin D. In the future, these trials should also include first-episode psychosis patients.

## Figures and Tables

**Table 1:** Demographic and clinical characteristics of the study samples.

	FEP (n=33)	MEP (n=33)	*P*
Age (years), mean (SD)	31.21 (10.03)	41.15 (9.88)	<0.001^†^
Gender, n (%)			
Male	23 (69.70%)	23 (69.70%)	1.000^‡^
Female	10 (69.30%)	10 (69.30%)	
Ethnicity, n (%)			
White	32 (96.97%)	32 (96.97%)	1.000^‡^
Black	1 (3.03%)	1 (3.03%)	
Education (years), mean (SD)	10.28 (3.40)		
Marital status, n (%)			
Single/divorced	7 (21.21%)	7 (21.21%)	1.000^‡^
Married/partner	26 (78.78%)	26 (78.78%)	
Blood collection season, n (%)			
Summer season	16 (48.48%)	11 (33.33%)	0.211^‡^
Winter season	17 (51.51%)	22 (66.66%)	
Tobacco, n (%)			
Yes	14 (42.42%)	19 (57.57%)	0.218^‡^
No	19 (57.57%)	14 (42.42%)	
Weight (kg), mean (SD)	70.86 (14.37)	74.86 (14.11)	0.257^†^
BMI (kg/m2), mean (SD)	23.85 (4.12)	25.08 (3.92)	0.221^†^
Diagnosis, n (%)			
Schizophrenia	12 (36.36%)	19 (57.58%)	0.148^§^
Delusional disorder	5 (15.15%)	2 (6.06%)	
Psychosis NOS	7 (21.21%)	1 (3.03%)	
Bipolar disorder	2 (6.06%)	4 (12.12%)	
Psychotic depression	4 (12.12%)	2 (6.06%)	
Cannabis induced psychosis	2 (6.06%)	3 (9.09%)	
Schizo-affective disorder	1 (3.03%)	2 (6.06%)	
Cannabis use, n (%)			
Yes	6 (18.18%)		
Duration of untreated psychosis (DUP) (days), mean (SD)	450.54 (952.64)		
Global Assessment Functioning (GAF), mean (SD)	59.21 (9.44)		
Medication Adherence Rating Scale (MARS), Mean (SD)	6.40 (2.14)		
Depressive symptom score (BDI), Mean (SD)	12.42 (9.10)		
Positive symptom subscale score (PANSS), Mean (SD)	12.84 (3.57)		
Negative symptom subscale score (PANSS), Mean (SD)	16.09 (7.27)		
General psychopathology subscale score (PANSS), Mean (SD)	31.59 (7.43)		

^†^Student's t-test

^‡^Chi-squared test

^§^Fisher's exact test

**Table 2:** Correlations between vitamin D levels and clinical variables in first-episode psychosis.

	25-Hydroxyvitamin D			
	Simple correlation		Multiple	
			linear regression	
	r	*P*	ß	*P*
Positive Symptoms	-0.165	0.358	0.045	0.793
Negative symptoms	-0.480	0.005*∗*	-0.269	0.178
General psychopathology	-0.569	0.001*∗*	-0.538	0.027*∗∗*
Depressive symptoms	-0.484	0.004*∗*	0.225	0.257

*∗*significant at p<0.01; *∗∗*significant at p<0.05.

## Data Availability

The SPSS data used to support the findings of this study are available from the corresponding author upon request.
